# Methylome and transcriptome analyses reveal insights into the epigenetic basis for the good survival of hypomethylated ER-positive breast cancer subtype

**DOI:** 10.1186/s13148-020-0811-1

**Published:** 2020-01-20

**Authors:** Xiao-Qiong Chen, Fan Zhang, Qi-Chen Su, Chi Zeng, Fu-Hui Xiao, Yu Peng

**Affiliations:** 1 State Key Laboratory of Biocatalysis and Enzyme Engineering of China, School of Life Sciences, Hubei University, Wuhan, 430062 China; 2 State Key Laboratory of Genetic Resources and Evolution, Kunming Institute of Zoology, Chinese Academy of Sciences, Kunming, 650223 China

**Keywords:** Breast cancer, Subtype, Methylation, Estrogen receptor, Survival probability

## Abstract

**Background:**

Breast cancer (BRCA) is a heterogeneous disease, characterized by different histopathological and clinical features and responses to various therapeutic measures. Despite the research progress of DNA methylation in classification and diagnosis of BRCA and the close relationship between DNA methylation and hormone receptor status, especially estrogen receptor (ER), the epigenetic mechanisms in various BRCA subtypes and the biomarkers associated with diagnostic characteristics of patients under specific hormone receptor status remain elusive.

**Results:**

In this study, we collected and analyzed methylation data from 785 invasive BRCA and 98 normal breast tissue samples from The Cancer Genome Atlas (TCGA) database. Consensus classification analysis revealed that ER-positive BRCA samples were constitutive of two distinct methylation subgroups; with the hypomethylated subgroup showing good survival probability. This finding was further supported by another cohort of ER-positive BRCA containing 30 subjects. Additionally, we identified 977 hypomethylated CpG loci showing significant associations with good survival probability in ER-positive BRCA. Genes with these loci were enriched in cancer-related pathways (e.g., Wnt signaling pathway). Among them, the upregulated 47 genes were also in line with good survival probability of ER-positive BRCA, while they showed significantly negative correlations between their expression and methylation level of certain hypomethylated loci. Functional assay in numerous literatures provided further evidences supporting that some of the loci have close links with the modulation of tumor-suppressive mechanisms via regulation gene transcription (e.g., *SFRP1* and *WIF1*).

**Conclusions:**

Our study identified a hypomethylated ER-positive BRCA subtype. Notably, this subgroup presented the best survival probability compared with the hypermethylated ER-positive and hypomethylated ER-negative BRCA subtypes. Specifically, we found that certain upregulated genes (e.g., *SFRP1* and *WIF1*) have great potential to suppress the progression of ER-positive BRCA, concurrently exist negative correlations between their expression and methylation of corresponding hypomethylated CpG loci. Therefore, our study indicates that different epigenetic mechanisms likely exist in ER-positive BRCA and provides novel clinical biomarkers specific to ER-positive BRCA diagnosis and therapy.

## Background

Breast cancer (BRCA) most frequently occurs in females, accounting for the greatest number of cancer-related deaths among women [1, 2]. Despite progress in BRCA therapy, the incidence of BRCA has continued to increase substantially [3] and remains a major public health burden worldwide [4]. BRCA is a complex heterogeneous disease [5, 6], containing about 20 morphologically distinct subtypes, such as inflammatory, pregnancy-associated, and comedo [7]. As different subtypes of BRCA exhibit distinct clinical outcomes and require subtype-specific treatment [6, 8, 9], the pathogenic mechanisms and diagnostic biomarkers under molecular-based BRCA subtypes were broadly concerned.

Recent years, transcriptional profile variations have been used to classify cancers into distinct subtypes [10], which are associated with different biological characteristics and clinical outcomes. Based on the expression status of several key genes, BRCA can be divided into at least four categories, including luminal A, luminal B, HER2-type, and basal-like [11]. For the time being, the crucial role of epigenetic mechanisms in modulating gene transcription has been of growing interest. Epigenetics involves the regulation of gene expression without changes in the genomic sequence [12]. To date, DNA methylation, a most studied epigenetic modification, is closely linked with tumorigenesis via gene expression modulation [13, 14]. In addition, evidence has revealed the difference in the molecular subtypes of certain cancer based on gene expression and DNA methylation [15]. Notably, a growing number of studies have shown that different BRCA subtypes display distinct methylation profiles [16–19]. The methylation status of certain CpG sites has been effectively used to evaluate the survival potential of BRCA [20]. Notably, hormone receptor, especially estrogen receptor (ER), is considered crucial factors leading to the different clinical outcomes of BRCA [21, 22]. Although abundant evidence has revealed the effects of hormone receptor on dynamic DNA methylation changes in the pathogenesis of BRCA [18, 23, 24], the DNA methylation signatures associated with the pathological development and diagnosis of BRCA under the context of certain hormone receptor have not yet been fully researched.

In the present study, we performed methylation-based BRCA classification using the Illumina Infinium Human Methylation450K dataset, which contained 785 invasive BRCA and 98 normal breast tissue samples from The Cancer Genome Atlas dataset (TCGA) using the UCSC Xena portal (https://xena.ucsc.edu/) [25]. In accordance with previous studies [18], our results strengthened current evidence supporting the crucial effects of hormone receptor (i.e., ER) on methylation-based clusters in BRCA. Interestingly, we identified two methylation-based subgroups in ER-positive BRCA and further determined that the hypomethylated subgroup presents a good survival probability, supported by another independent dataset of ER-positive BRCA. Moreover, the hypomethylated loci in this subgroup may play roles to suppress tumor progression via promoting the transcription of putative suppressor genes in ER-positive BRCA subgroup.

## Methods

### Data collection and pre-processing

The methylation dataset of samples and associated clinical information were obtained TCGA database using the UCSC Xena platform (https://xena.ucsc.edu/) [25]. Based on the “01” and “11” barcodes in TCGA, we collected a dataset containing 785 BRCA and 98 normal breast tissue samples detected by the Illumina Infinium HumanMethylation450 BeadChips platform, which covered 485,577 CpG loci. The methylation levels of the CpG sites in each sample were represented as a *β* value ranging from 0 to 1. We first filtered the CpG sites with any missing values across the samples and retained 363,870 CpG sites for subsequent analysis. In addition, the sample-matched RNA-seq data, which quantified the gene expression value by log2(x + 1) transformed RSEM (RNA-Seq by Expectation-Maximization) normalized count, was also downloaded from TCGA using same data portal. In this study, the methylation and expression values were normalized by the ‘normalize.quantiles’ function in R package “preprocessCore”. Another methylation dataset of BRCA was collected from NCBI’s Gene Expression Omnibus (GEO) portal with accession number GSE37754, in which only the 30 ER-positive tumor samples were considered. Moreover, 124 samples from 5 different types of normal tissues with methylation and RNA-Seq datasets were downloaded from TCGA (https://xena.ucsc.edu/).

### Consensus cluster analysis

Initially, we extracted 18,870 most informative (variable) CpG sites using a standard deviation (SD) threshold larger than 0.2 in the tumor samples. To perform methylation-based consensus classification of BRCA, we utilized the R package “ConsensusClusterPlus”, which provides quantitative and visual stability evidence for estimating the number of unsupervised clusters in a dataset [26]. In the clustering processes, a k-means algorithm and 1-Pearson correlation distance metric were used. To obtain stable clusters, we performed 100 iterations with a parameter of maxK = 20. In each iteration, 80% of tumor samples were sampled.

### Gene annotation and enrichment analysis

Gene annotation analysis of the CpG sites was obtained in the data descriptions of Illumina HumanMethylation450 BeadChips (GPL13534) from the National Center for Biotechnology Information (NCBI, https://www.ncbi.nlm.nih.gov/). Gene set enrichment analysis was performed using web tool Metascape [27]. Protein-protein interaction networks were constructed using STRING (v11.0) with default parameters (https://string-db.org/) [28].

### Statistical analysis

The statistical analyses were performed on the R platform (https://cran.r-project.org/). We analyzed the CpG sites with methylation differences between the two groups using Student’s *t* tests and performed the methylation-gene expression correlation analysis using Pearson correlation coefficients. For survival analyses in 10 years’ interval, we used the “survival” package with the cox model in R using sample data, including death or last follow-up, from TCGA. When calculating the association of the methylation status of CpG sites and survival probability, we considered the samples with mean methylation level greater than 75% quantile as hypermethylated subgroup while those with value lesser than 25% quantile as hypomethylated subgroup. We drew the heatmaps and survival curves using the “pheatmap” and “survminer” packages, respectively.

## Results

### Identification of two major DNA methylation-based clusters in BRCA

We collected methylation data screened by Infinium HumanMethylation450 BeadChips (covering 485,577 CpG loci) from the TCGA database and analyzed a total of 785 BRCA and 98 normal breast tissue samples. After removing the CpG sites with missing methylation *β* values in the 883 samples, 363,870 CpG sites were retained. We then set a threshold of tumor methylation variation with a standard deviation (SD) > 0.2 and extracted 18,870 CpG sites for subsequent analyses.

**Fig. 1:**
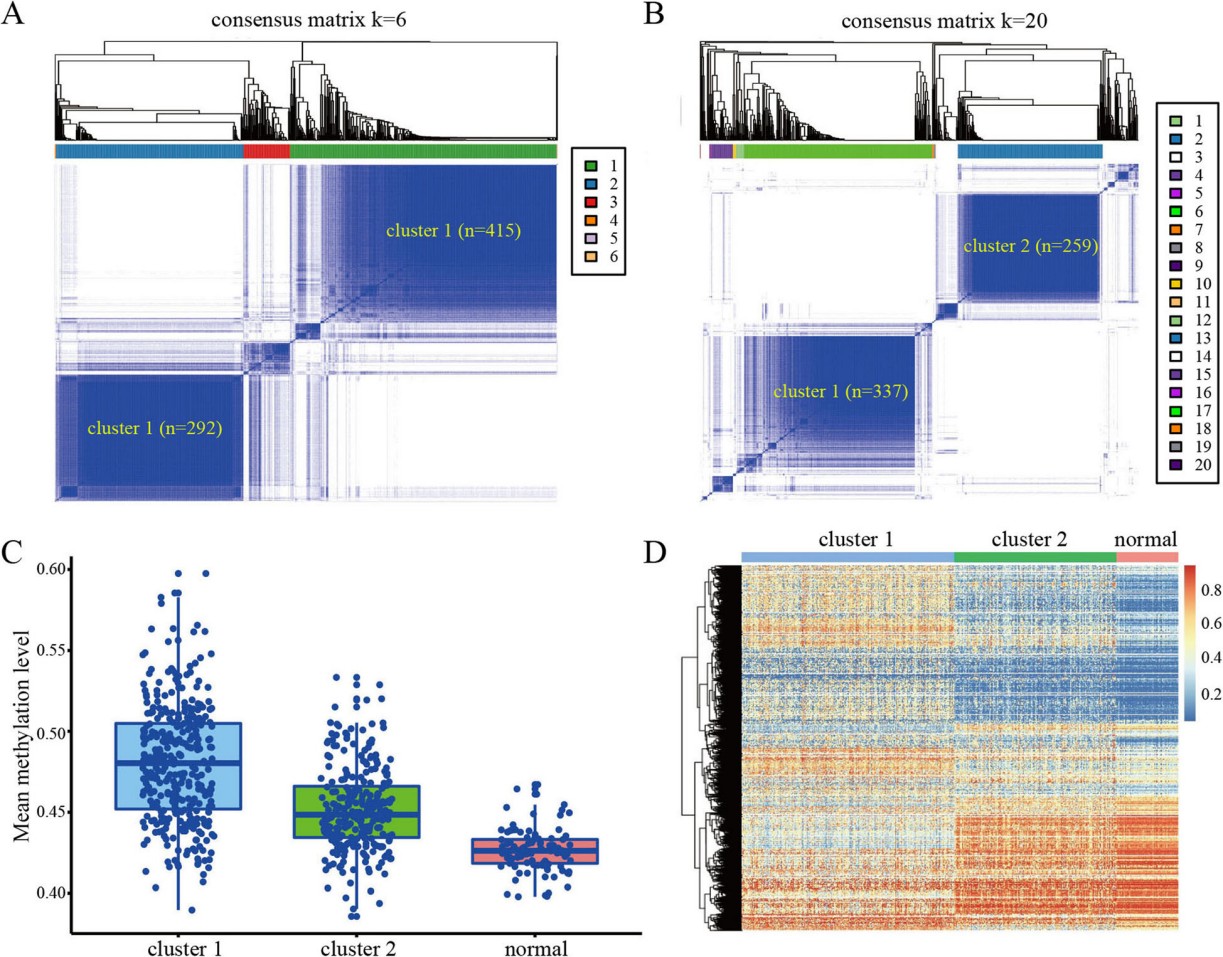
DNA methylation-based clusters of BRCA samples. **a** Consensus clustering of BRCA samples with *K* = 6. **b** Consensus clustering of BRCA samples with *K* = 20. **c** Mean methylation levels of cluster 1, cluster 2, and normal samples. **d** Heatmap of BRCA methylation differences between cluster 1, cluster 2, and normal samples

To determine the methylation-based clusters of BRCA, we performed unsupervised hierarchical cluster analysis of the cancer samples using 1-Pearson correlation distance with the ‘ConsensusClusterPlus’ package [25]. When setting the cluster threshold to *K* = 6, we identified two major clusters that contained 90.0% of all cancer samples, with 415 and 292 samples in cluster 1 and cluster 2, respectively (Fig. 1a). We then utilized a stringent threshold of *K* = 20 and found that most samples in the two clusters remained stable, retaining 337 (81.2%, 337/415) and 259 (88.7%, 259/292) samples in cluster 1 and cluster 2, respectively (Fig. 1b). In addition, we calculated the average methylation levels of the 18,504 CpG sites in cluster 1, cluster 2, and normal samples. Compared with the normal samples, the samples in cluster 1 had the highest methylation score, whereas those in cluster 2 had an intermediate methylation score (Fig. 1c). The heatmap also revealed markedly distinct methylation profiles between clusters 1 and 2 of the BRCA samples (Fig. 1d).

### A hypomethylated ER-positive BRCA subgroup with good survival probability

**Fig. 2:**
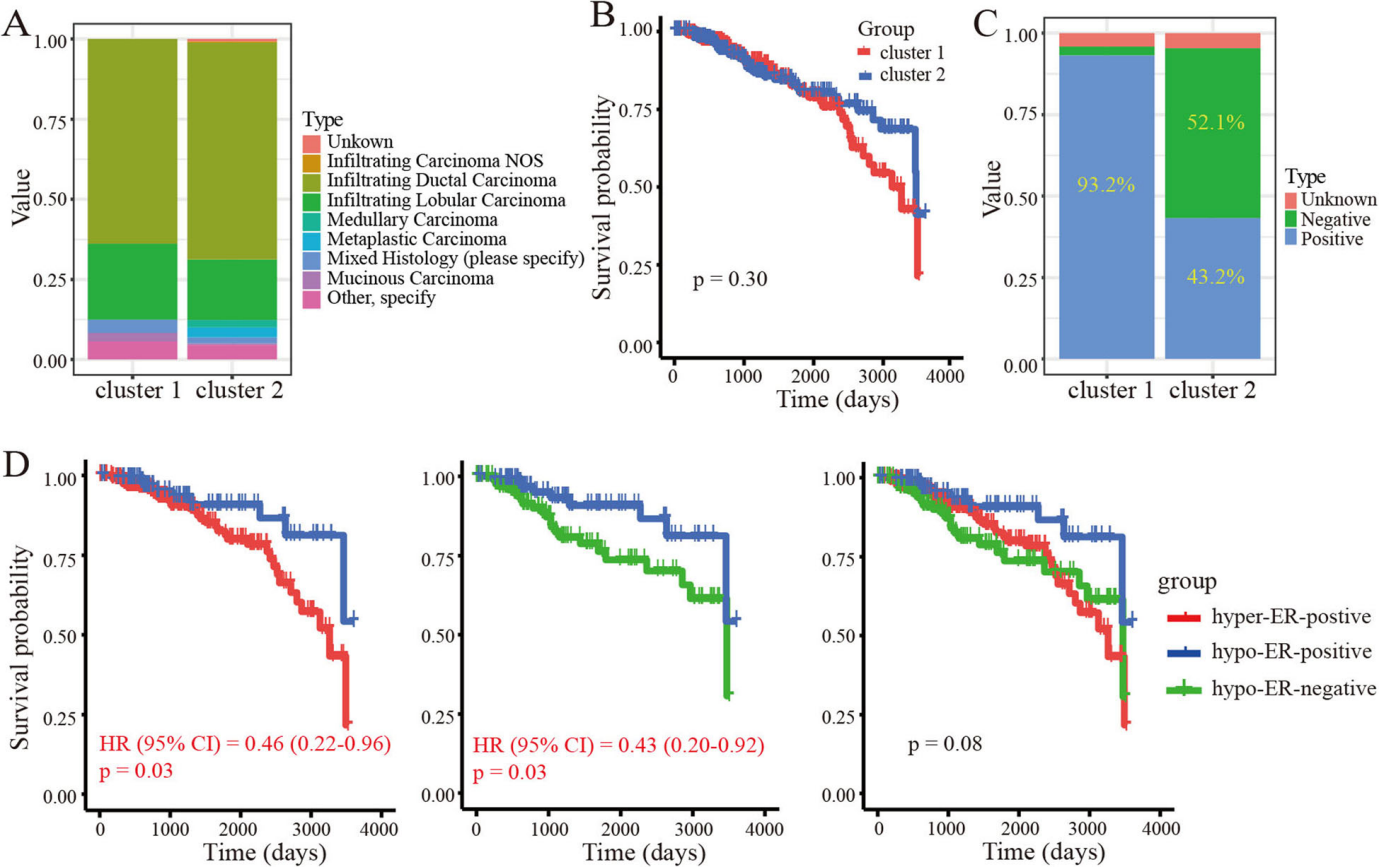
Clinical signatures of clusters in BRCA samples. **a** Histological types of BRCA in cluster 1 and cluster 2. **b** Kaplan-Meier survival curve of BRCA in cluster 1 and cluster 2. **c** Estrogen receptor status of BRCA in cluster 1 and cluster 2. **d** Kaplan-Meier survival curve of BRCA in hyper-ER-positive, hypo-ER-positive and hypo-ER-negative subgroups

We next investigated the histological types (e.g., infiltrating ductal carcinoma, infiltrating lobular carcinoma) of the BRCA samples in clusters 1 and 2. Results showed that the two clusters exhibited similar histological composition ratios (Fig. 2a). In addition, we carried out a survival analysis to explore the overall survival probability of BRCA in clusters 1 and 2. No significant survival differences were found between the two clusters (*p* = 0.30) (Fig. 2b).

As hormone receptor status, especially ER, has a close relationship with tumor progression and methylation modification [24, 29], we explored the status of ER in clusters 1 and 2 based on the information recorded in the clinical dataset from TCGA. Results showed that most (93.2%; 314/337) samples in cluster 1 were ER-positive BRCA. Interestingly, the samples in cluster 2 were more complex and included 43.2% (112/259) ER-positive BRCA and 52.1% (135/259) ER-negative BRCA (Fig. 2c). Similarly, the overall methylation level of ER-positive tumors in cluster 2 found to be lower than that of ER-positive tumors in cluster 1 (Additional file 1: Figure S1). Thus, according to the hormone receptor status and methylation profiles, we divided the tumors into three subgroups, namely hypermethylated ER-positive BRCA (hyper-ER-positive), hypomethylated ER-positive BRCA (hypo-ER-positive), and hypomethylated ER-negative BRCA (hypo-ER-negative). We then analyzed the survival probability in the three subgroups and found a significantly good survival probability for hypo-ER-positive BRCA compared with hyper-ER-positive and hypo-ER-negative BRCA (*p* < 0.05) (Fig. 2d).

We then investigated the distributions of PAM50 gene signature-based BRCA subtypes (e.g., luminal A and luminal B) on these 3 methylation-based subgroups [30]. Results showed that hypo/hyper-ER-positive BRCA subgroups contain a number of luminal A and luminal B tumors, while the hypo-ER-negative subgroup mainly consists of basal tumors (Additional file 1: Figure S2A). Here, we focused on the ER-positive BRCA patients and found that the hypo-ER-positive subgroup contains a small number of luminal B breast cancers compared with that in the hyper-ER-positive subgroup (9.8% vs. 27.4%), echoing the fact of worse survival for luminal B BRCA patients (Additional file 1: Figure S2B). In addition, we also observed a similar distribution pattern for the progesterone receptor (PR) status in the methylation-based BRCA subgroups (Additional file 1: Figure S2C). These findings indicated a complex relationship in subtyping the BRCA patients based on gene expression and DNA methylation, which has been observed in other tumors [15].

### A similar observation in another independent population

**Fig. 3:**
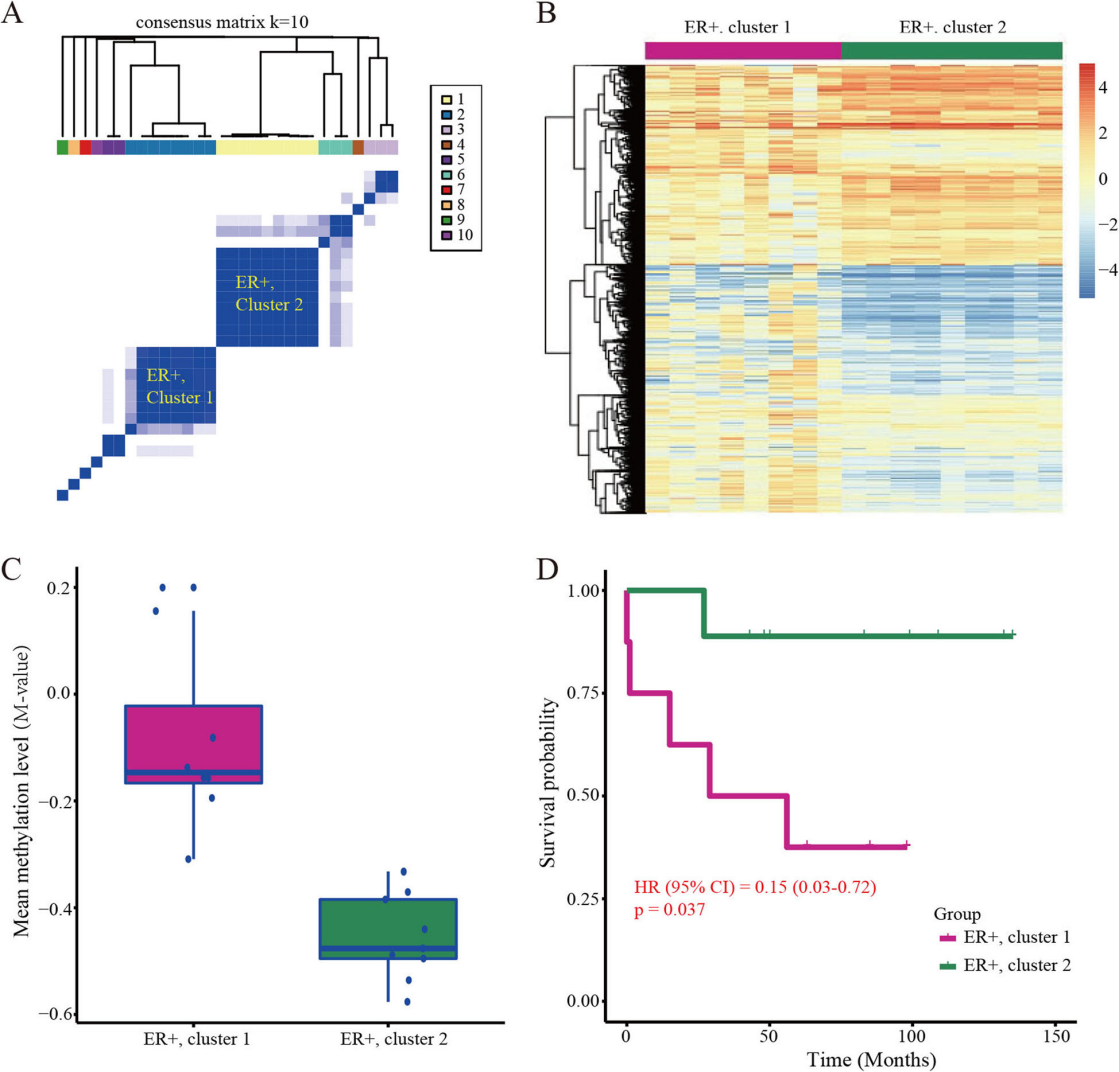
Consensus clustering analysis of BRCA in an independent population. **a** Consensus clustering of ER-positive BRCA with *K* = 10. **b** Heatmap plot of methylation differences of ER-positive cancers in cluster 1 and cluster 2. **c** Mean methylation level of the two major BRCA subgroups. **d** Kaplan-Meier survival curve of the two subtyping ER-positive cancers

To test whether our findings can be observed in other populations, we collected and analyzed another methylation dataset containing 30 ER-positive BRCA samples. Similarly, consensus clustering analysis still divided the cancer samples into two major subgroups (Fig. 3a), which were confirmed by the heatmap plot (Fig. 3b). Consistently, further analysis showed that the hypomethylated ER-positive BRCA subgroup (i.e., ER+, cluster 2) indeed presents a good survival probability (Fig. 3c, d).

### Abundant CpG sites with methylation differences between hypo-ER-positive and hyper-ER-positive BRCA subtypes

**Fig. 4:**
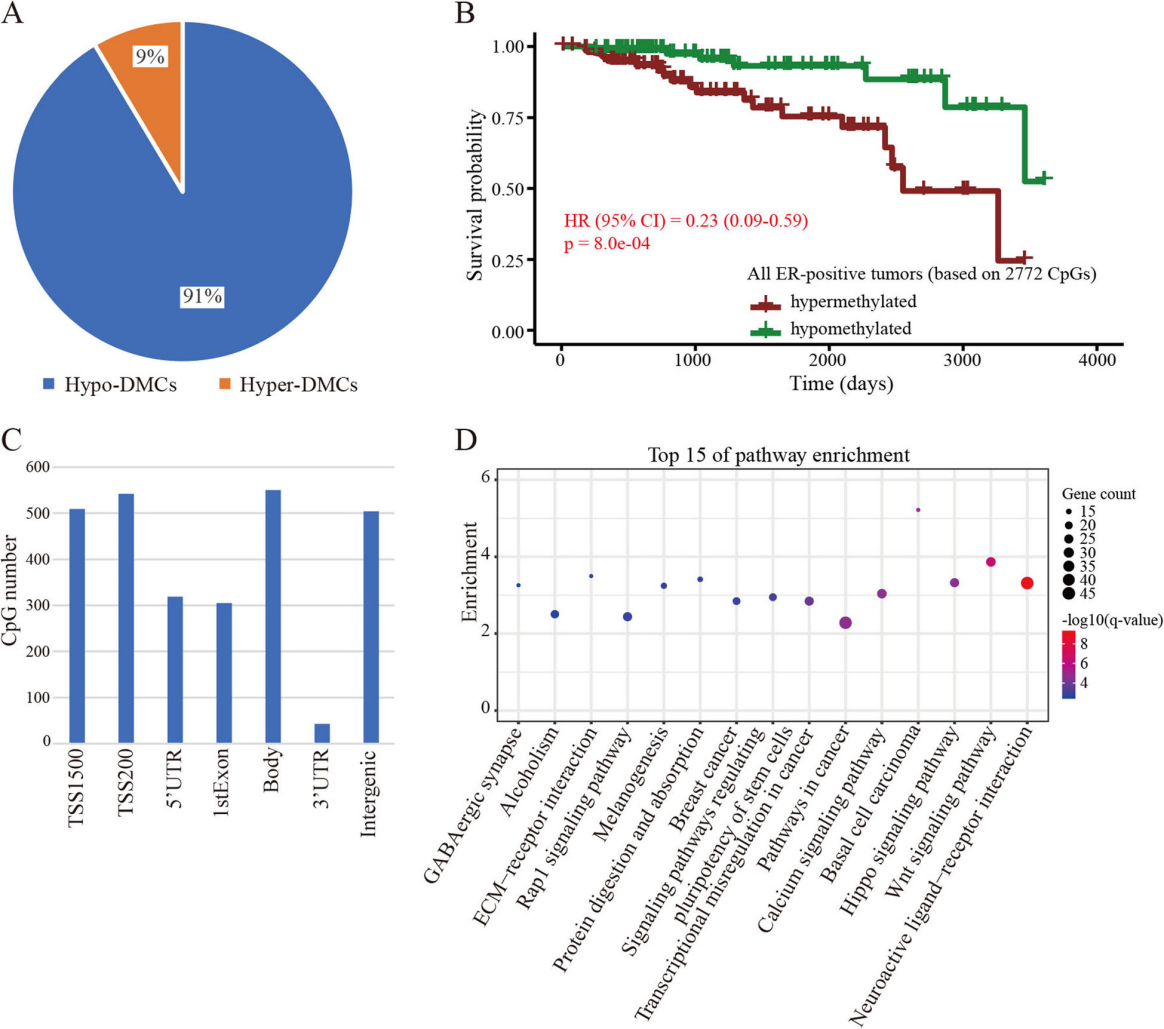
CpG sites with methylation differences between the two ER-positive BRCA subgroups. **a** Percentage of hypermethylated and hypomethylated sites between hypo-ER-positive and hyper-ER-positive cancer subgroups. **b** Survival curve in the two subgroups based on the methylation level of the 2772 hypo-DMCs. **c** Distribution of hypo-DMCs in various genomic sequences. **d** Genes with hypo-DMCs enriched in pathways related with cancer

As the ER-positive BRCA had two methylation-based subgroups showing a significant difference in survival probability, we focused on those CpG sites that contributed to the classification of ER-positive BRCA. Using a threshold BH-adjusted *p* value <  0.01 and |log2 (fold-change)| > 1, we identified 3032 differentially methylated CpG sites (DMCs) between the hyper-ER-positive and hypo-ER-positive BRCA subgroups. Among these DMCs, approximately 91.4% (2772/3032) were hypomethylated in the hypo-ER-positive subgroup (Fig. 4a). Then, we also divided the ER-positive BRCA into two subgroups based on the mean methylation of the 2772 hypomethylated DMCs (hypo-DMCs; see “Materials and methods”). The survival analysis further revealed that the ER-positive cancer samples with low methylation content have a good survival probability (*p* = 0.0008) (Fig. 4b and (Additional file 1: Figure S3A). Whereas, no significant association was observed between the methylation content of these hypo-DMCs and the survival of hypo-ER-negative BRCA subgroup (Additional file 1: Figure S3B).

According to the gene annotation analysis, the hypo-DMCs were located on the TSS1500 (509), TSS200 (542), 5’UTR (319), 1stExon (305), Body (550), 3’UTR (43), and intergenic regions (504) (Fig. 4c). In addition, most hypo-DMCs (94.5%; 2619/2772) were located on regions associated with CpG islands (Additional file 1: Figure S4). To explore the roles of the hypo-DMCs in tumorigenesis, we performed gene set enrichment analysis. Results showed that 1190 genes associated with the hypo-DMCs were significantly enriched in pathways, such as Wnt (hsa04310), Hippo (has04309), Rap1 (hsa04015), basal cell carcinoma (hsa05217), transcriptional misregulation in cancer (hsa05202), and breast cancer (hsa05224) (*q* < 0.05) (Fig. 4d). These pathways indeed play important roles in cancer progression [31–33], indicating the potential roles of these hypo-DMCs in inhibiting ER-positive BRCA progression.

### Tumor suppression by key hypo-DMCs in ER-positive BRCA subgroup

To further confirm the potential tumor-suppressing roles of the hypo-DMCs, we reanalyzed the association between methylation level and survival probability. Among them, 979 CpG loci have significant associations between methylation level and survival in ER-positive BRCA (*p* < 0.05). As expectedly, 977 loci present the relationship between hypomethylation and good survival probability, with the hazard ratio (HR) lesser than 0.48 (Additional file 1: Figure S5A). Similarly, genes with these loci were still enriched in cancer-related pathways, such as Wnt, Rap1, microRNA in cancer and transcriptional misregulation in cancer (*q* < 0.05) (Additional file 1: Figure S5B). We then performed multivariate cox model analysis to correct the effects of covariates (i.e., hormone receptor, histological type, pathological stage, and PAM50 gene signatures based subtype) and found that 68.3% (667/977) of the loci still display significant associations between their methylation level and survival of BRCA patients.

**Fig. 5:**
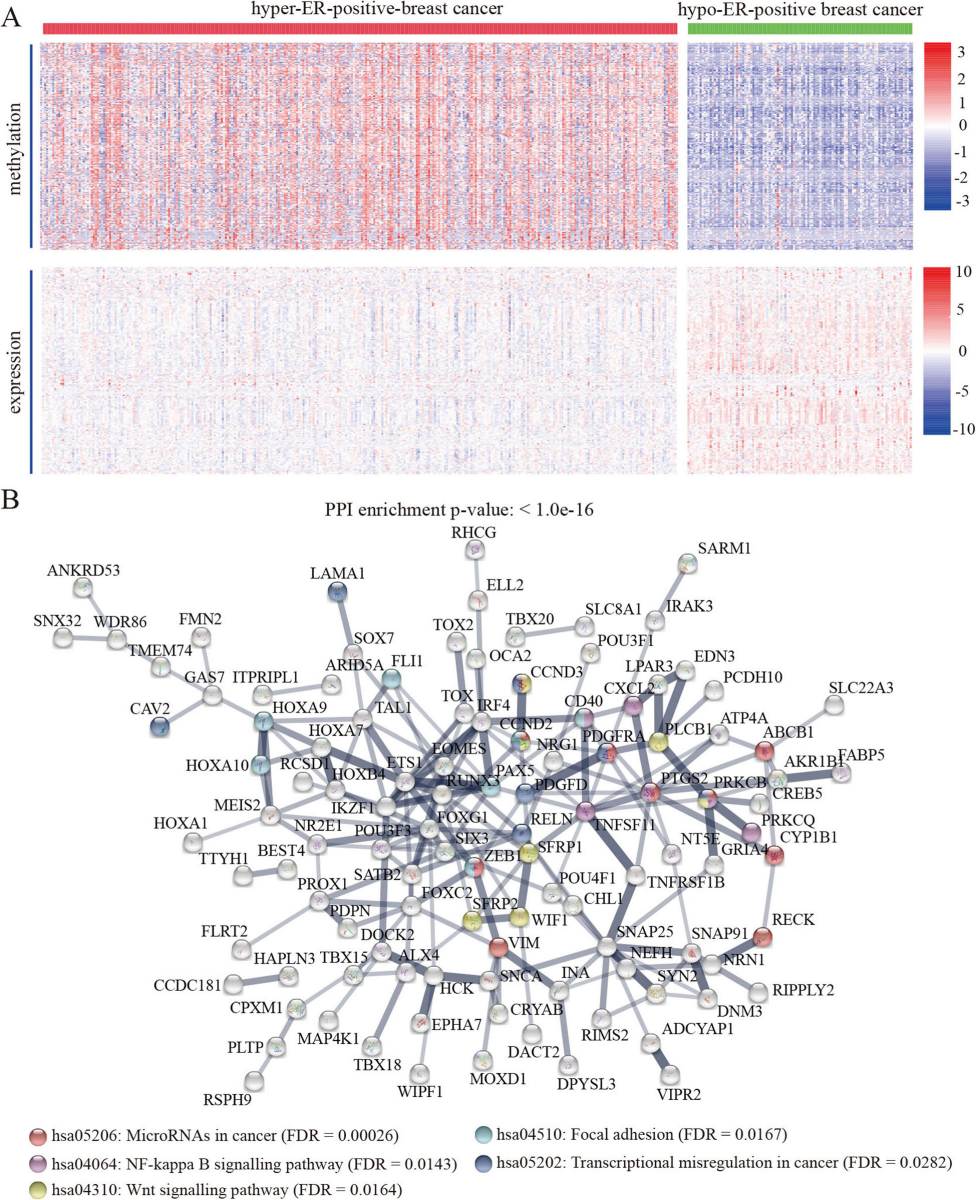
Hypo-DMCs showing significant associations between hypomethylation and good survival probability in ER-positive BRCA. **a** Heatmap of upregulated genes with negative associations between their expression and methylation of the hypo-DMCs. **b** Protein-protein interaction network of genes with the hypo-DMCs

To test whether the hypo-DMCs have the potential to promote transcription of genes with tumor-suppressing roles, we performed methylation and expression correlation analysis and identified a total of 178 genes that displaying negative associations between their expression and methylation of certain hypo-DMCs (Fig. 5a). Further protein-protein interaction analysis showed that the proteins have significantly more interactions than expected (Fig. 5b). Similarly, the proteins in the network were also enriched in pathways including NF-kappa B, Wnt, microRNAs in cancer, focal adhesion and transcriptional misregulation in cancer (FDR < 0.05) (Fig. 5b).

**Fig. 6:**
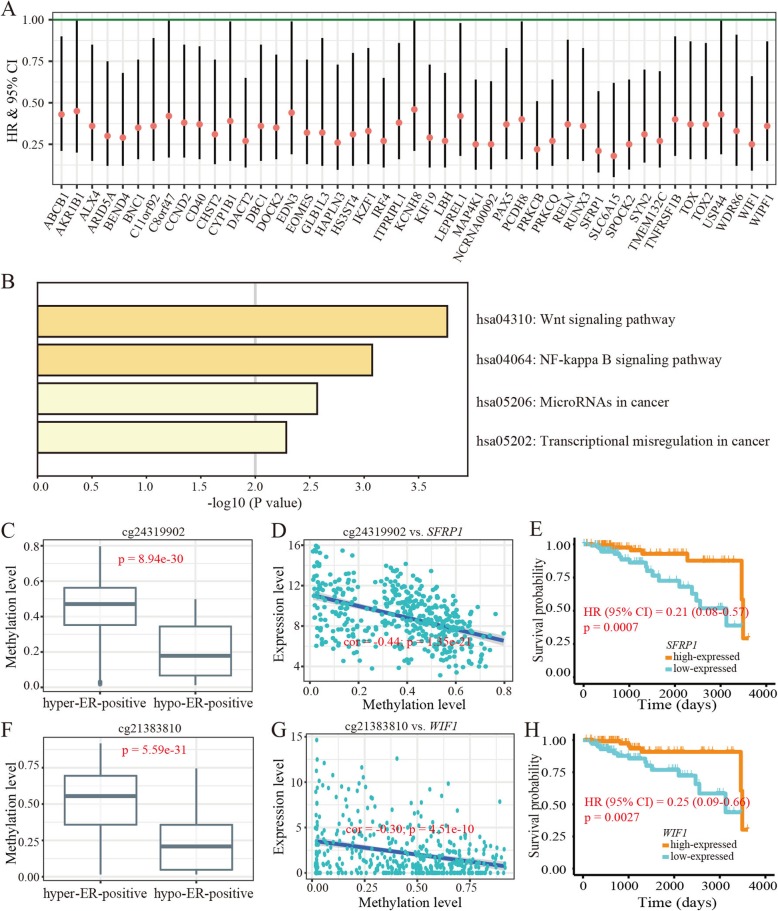
Epigenetic activation of putative tumor suppressor genes in improving survival of ER-positive BRCA. **a** Forest plot for assesses of the 47 genes on survival in ER-positive BRCA (bars represent the 95% CI). **b** KEGG pathways enriched by the 47 genes. **c** Methylation differences in cg24319902 between hypo-ER-positive and hyper-ER-positive BRCA subgroups (*p* < 0.05). **d** Association between methylation level of cg24319902 and expression level of *SFRP1*. **e** Survival plot of *SFRP1* expression with survival in ER-positive cancer. **f** Methylation differences in cg21383810 between hypo-ER-positive and hyper-ER-positive cancer (*p* < 0.05). **g** Association between methylation level of cg21383810 and expression level of *WIF1*. **h** Survival plot of *WIF1* expression with survival in ER-positive cancer

We then analyzed the correlation between the expression level of genes and survival probability of ER-positive BRCA and identified 47 (47/178) genes with high expression and good survival probability (Fig. 6a and (Additional file 1: Table S1). Consistently, these 47 genes were enriched in pathways including Wnt signaling pathway, NF-kappa B signaling pathway, microRNA in cancer and transcriptional misregulation in cancer (*p* < 0.05) (Fig. 6b).

## Discussion

BRCA is classified into distinct subtypes by various molecular features, with consequently different treatment strategies [34, 35]. Abundant research has revealed that hormone receptor (i.e., ER) status is one of the most important features of BRCA [36, 37]. Epidemiological research has shown that about 70% of BRCA patients are ER-positive [38]. Nevertheless, ER-positive BRCA still constitutes a more heterogeneous group for diagnosis and treatment [39, 40]. For instance, according to the HER2 status and KI67 protein level, ER-positive BRCA also consists of two major subtypes, i.e., luminal A and luminal B, with luminal B tumors presenting a worse prognosis than luminal A subtype [35, 40]. Currently, accumulating evidence has shown that epigenetic modifications, in especial DNA methylation, play important roles in BRCA progression and present close associations with the BRCA heterogeneity [41, 42]. Therefore, the therapeutic targets and biomarkers on epigenetic layers specific to molecular-based BRCA subclasses (e.g., ER-positive) remain to be investigated.

In the present study, we identified a hypomethylated ER-positive BRCA subgroup, with a considerable number of samples displaying similar methylation levels as ER-negative BRCA. More remarkably, this subgroup presented the best survival probability compared with the hyper-ER-positive and hypo-ER-negative BRCA. This finding is in contrast to the general view that ER-positive BRCA always exhibits higher methylation and better survival than that of ER-negative BRCA [18, 29].

Additionally, we identified hundreds of hypo-DMCs that showing significant associations between hypomethylation and good survival in the hypo-ER-positive BRCA. Indeed, further analysis revealed that the genes with these sites preferentially locate on cancer-related pathways, such as the Wnt and Rap1 signaling pathways [31, 32]. These findings imply that the hypo-DMCs play crucial roles in suppressing tumor progression via regulation of gene activity in pathways associated with ER-positive BRCA progression. Moreover, our observations were supported by the survival analysis of certain DMCs and corresponding genes in the ER-positive BRCA. By scanning the literatures associated function of corresponding genes, some cases indeed supported the roles of hypo-DMCs in inhibition of tumor promotion by regulating gene transcription. For example, cg24319902 is a CpG site located in the TSS200 region of *SFRP1*, loss expression of which is reported to be associated with the progression of BRCA [43]. The methylation level of this CpG site decreased in hypo-ER-positive BRCA subgroup compared with that in hyper-ER-positive BRCA subgroup (Fig. 6c). Due to the negative correlation between its methylation and the expression level of *SFRP1* (Fig. 6d and (Additional file 1: Figure S6A), the hypomethylation of cg21790626 likely reduced the severity of BRCA in hypo-ER-positive subgroup. Indeed, the higher expression level of *SFRP1* was associated with a good survival of ER-positive cancers in the TCGA dataset (Fig. 6e). In addition, cg21383810 was also hypomethylated in hypo-ER-positive BRCA subgroup (Fig. 6f). This CpG site, located in the TSS1500 region of *WIF1*, was found to be negatively associated with the expression of *WIF1* (Fig. 6g and (Additional file 1: Figure S6B). Given that the contribution of epigenetic silencing *WIF1* to breast tumorigenesis as reported in the previous study [44], it is likely that the loss of methylation in the hypo-ER-positive cancer samples will suppress disease acceleration, which was also supported by the association between its expression and patients’ chances of survival (Fig. 6h). Taken together, our study indicates that the hypomethylation of ER-positive BRCA in some CpG loci has contributions to the modulation of certain tumor suppression mechanisms. This research provides potential epigenetic diagnostic biomarkers and therapeutic targets specific to ER-positive BRCA. And yet for that, further clinical and experimental studies will facilitate the extending of these findings.

## Supplementary information

**Additional file 1: Figure S1.** Mean methylation level of BRCA samples in cluster 1: ER-positive, cluster 2: ER-positive, and cluster 2: ER-negative. **Figure S2.** Relationship of the PAM50 gene signatures and methylation-based BRCA subgroups. **Figure S3.** Survival analysis based on the 2722 hypo-DMCs. **Figure S4.** Distribution of sites in regions related to CpG islands. **Figure S5.** The 977 loci with relationships between hypomethylation and good survival probability of ER-positive breast cancer. **Figure S6.** Methylation-gene expression associations for the cg24319902-*SFRP1* and cg21383810-*WIF1* pairs in normal tissues. **Table S1.** Information of hypo-DMC activating genes that presenting a significant association between high expression and good survival probability in ER-positive breast cancer.

## Abbreviations

BH: Benjamini-Hochberg
BRCA: Breast cancer
CI: Confidence interval
DMC: Differentially methylated CpG site
ER: Estrogen receptor
HR: Hazard ratio
RSEM: RNA-Seq by Expectation-Maximization
SD: Standard deviation
SFRP1: Secreted frizzled-related protein 1
TCGA: The Cancer Genome Atlas
WIF1: WNT inhibitory factor 1
