# Supplementary material for: Methylome and transcriptome analyses reveal insights into the epigenetic basis for the good survival of hypomethylated ER-positive breast cancer subtype
Source: Clin Epigenetics. 2020 Jan 20;12:16. doi: 10.1186/s13148-020-0811-1 (PMC6971951; doi:10.1186/s13148-020-0811-1)
Supplement: Supplementary file 1 — Additional file 1: Figure S1. Mean methylation level of BRCA samples in cluster 1: ER-positive, cluster 2: ER-positive, and cluster 2: ER-negative. Figure S2. Relationship of the PAM50 gene signatures and methylation-based BRCA subgroups. Figure S3. Survival analysis based on the 2722 hypo-DMCs. Figure S4. Distribution of sites in regions related to CpG islands. Figure S5. The 977 loci with relationships between hypomethylation and good survival probability of ER-positive breast cancer. Figure S6. Methylation-gene expression associations for the cg24319902-SFRP1 and cg21383810-WIF1 pairs in normal tissues. Table S1. Information of hypo-DMC activating genes that presenting a significant association between high expression and good survival probability in ER-positive breast cancer. [file 13148_2020_811_MOESM1_ESM.doc]

**Supplemental Information for “*****Methylome and transcriptome analyses reveal insights into the epigenetic basis for the good survival of hypomethylated ER-positive breast cancer subtype*”**

**Index:**

**Figure S1.** Mean methylation level of BRCA samples in cluster 1: ER-positive, cluster 2: ER-positive, and cluster 2: ER-negative.

**Figure S2.** Relationship of the PAM50 gene signatures and methylation based BRCA subgroups. (A) Distribution of gene expression based BRCA subtypes within the methylation-based BRCA subgroups. (B) Survival curve of the luminal A and luminal B BRCA subtypes. (C) Distribution of the PR status in the two ER-positive BRCA subgroups.

**Figure S3.** Survival analysis based on the 2,722 hypo-DMCs. (A) Survival curve based on the mean methylation level of these loci hypo-ER-positive breast cancer subgroup. (B) Survival curve based on the mean methylation level of these loci in hypo-ER-negative breast cancer subgroup.

**Figure S4.** Distribution of sites in regions related to CpG islands.

**Figure S5.** The 977 loci with relationships between hypomethylation and good survival probability of ER-positive breast cancer. (A) Hazard ratio of the 977 loci with survival probability. (B) Pathway enrichments of the genes corresponding to these loci.

**Figure S6.** Methylation-gene expression associations for the cg24319902-*SFRP1* and cg21383810-*WIF1* pairs in normal tissues.

**Table S1.** Information of hypo-DMC activating genes that presenting a significant association between high expression and good survival probability in ER-positive breast cancer.

**
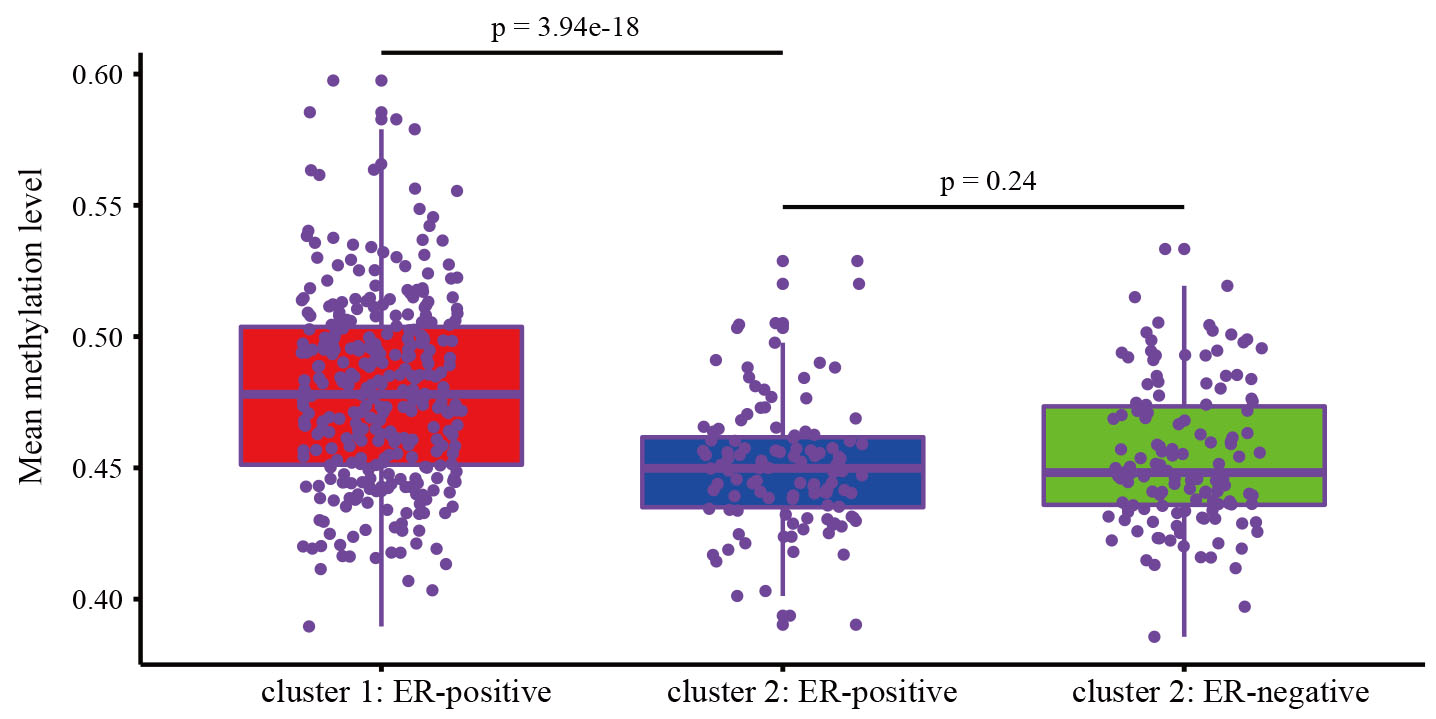
**

**Figure S1. Mean methylation level of BRCA samples in cluster 1: ER-positive, cluster 2: ER-positive, and cluster 2: ER-negative.**


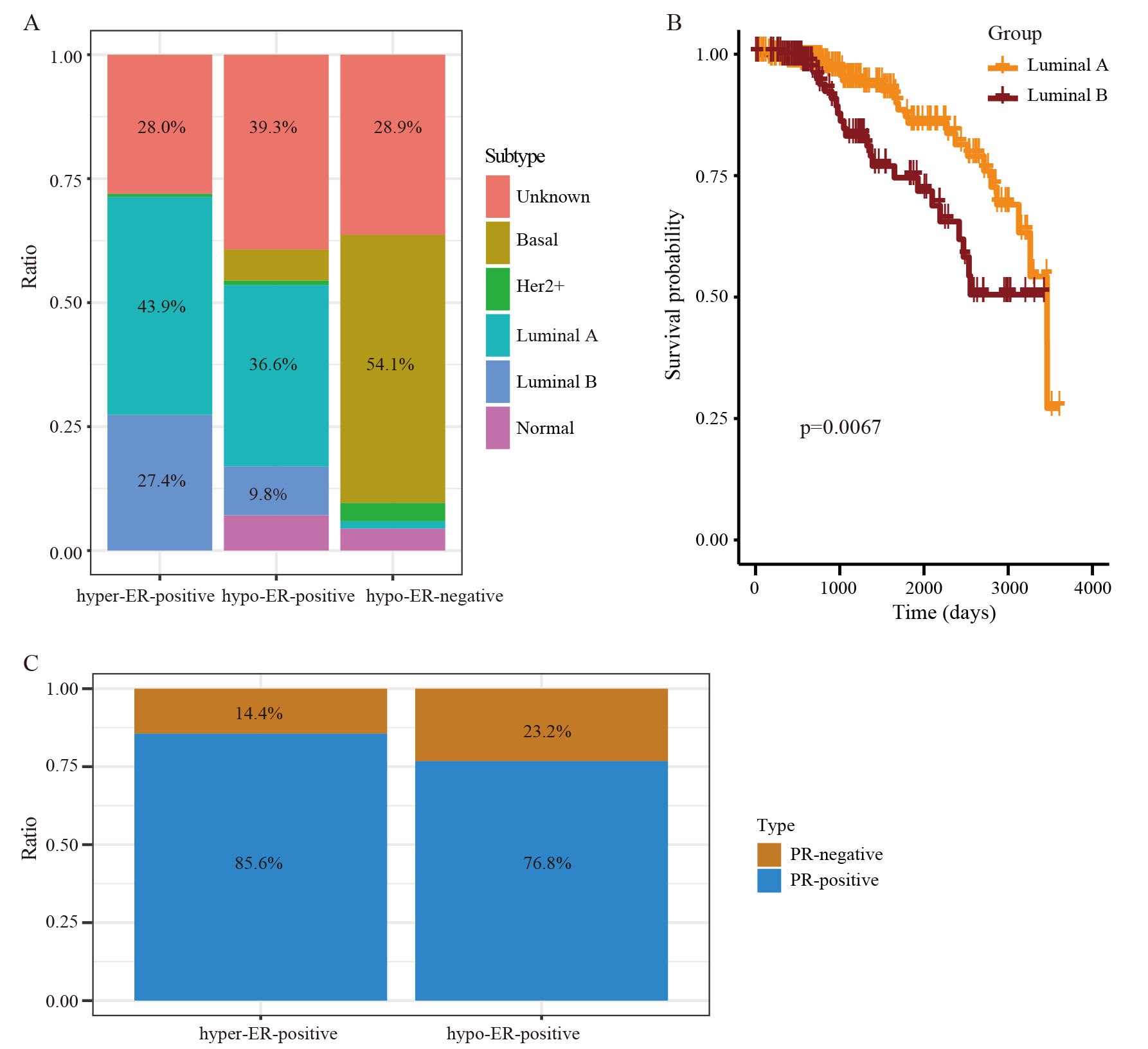


**Figure S2. Relationship of the PAM50 gene signatures and methylation based BRCA subgroups.** (A) Distribution of gene expression based BRCA subtypes within the methylation-based BRCA subgroups. (B) Survival curve of the luminal A and luminal B BRCA subtypes. (C) Distribution of the PR status in the two ER-positive BRCA subgroups.


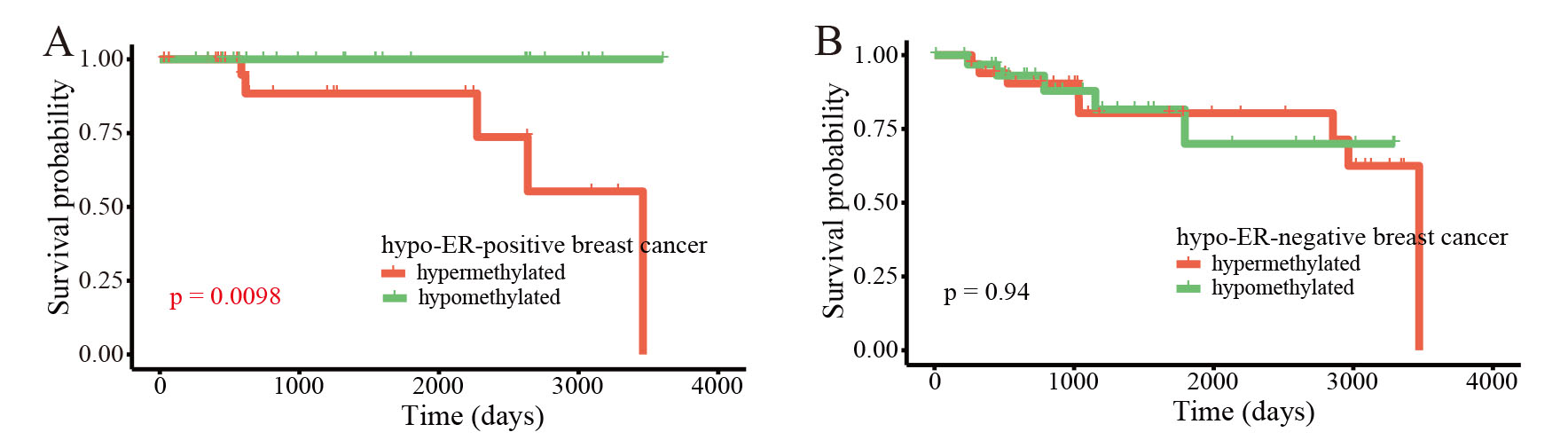


**Figure S3. Survival analysis based on the 2,722 hypo-DMCs.** (A) Survival curve based on the mean methylation level of these loci hypo-ER-positive breast cancer subgroup. (B) Survival curve based on the mean methylation level of these loci in hypo-ER-negative breast cancer subgroup.


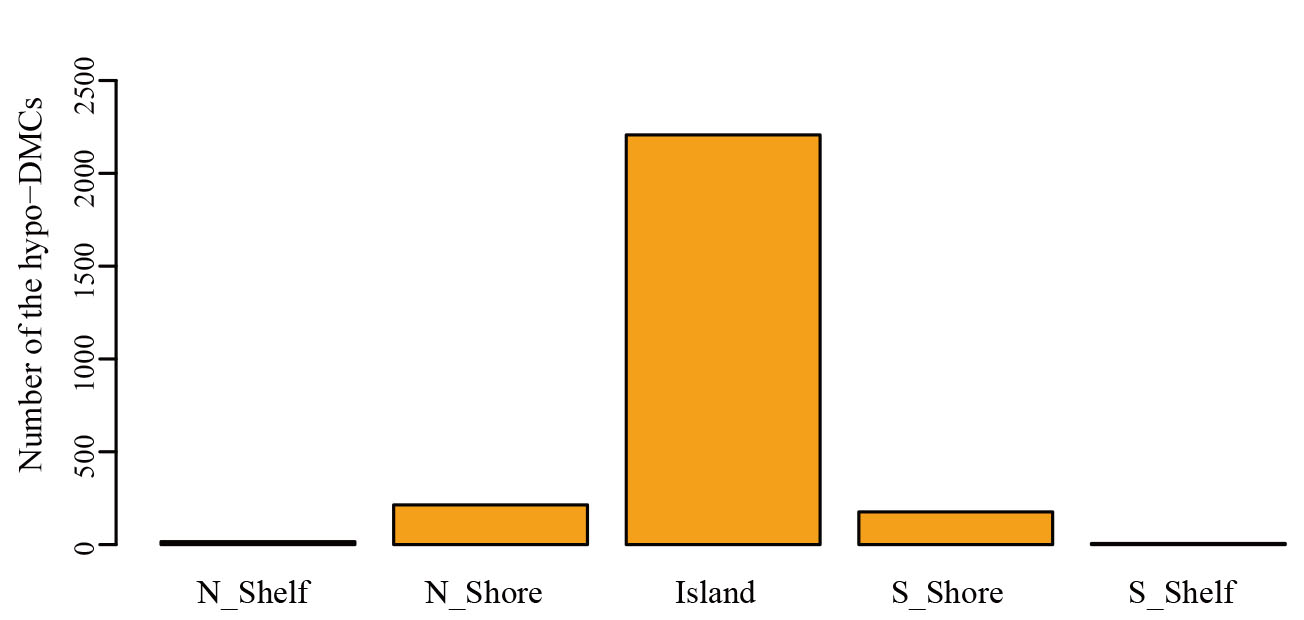


**Figure S4. Distribution of sites in regions related to CpG islands.**


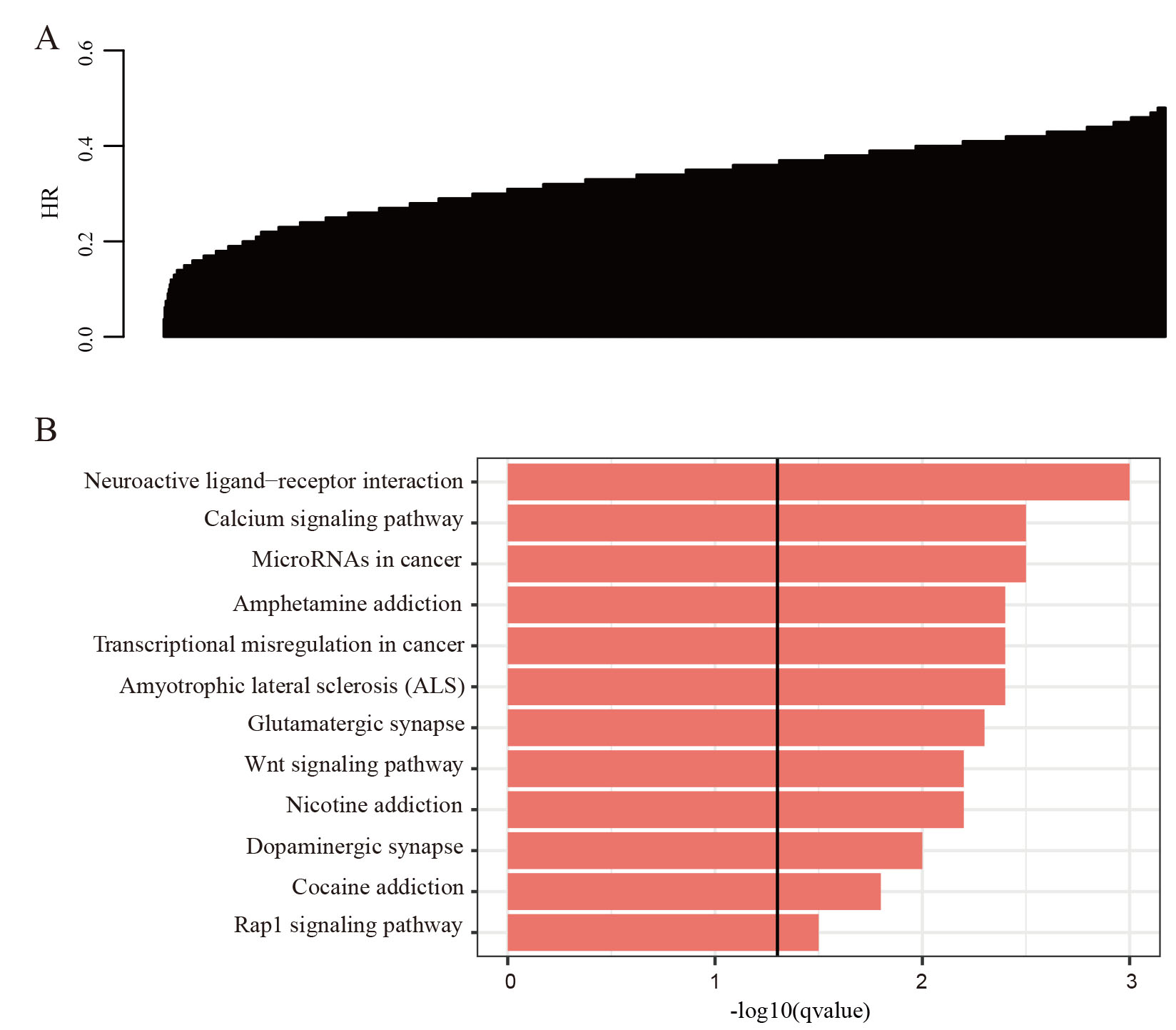


**Figure S5. The 977 loci with relationships between hypomethylation and good survival probability of ER-positive breast cancer.** (A) Hazard ratio of the 977 loci with survival probability. (B) Pathway enrichments of the genes corresponding to these loci.


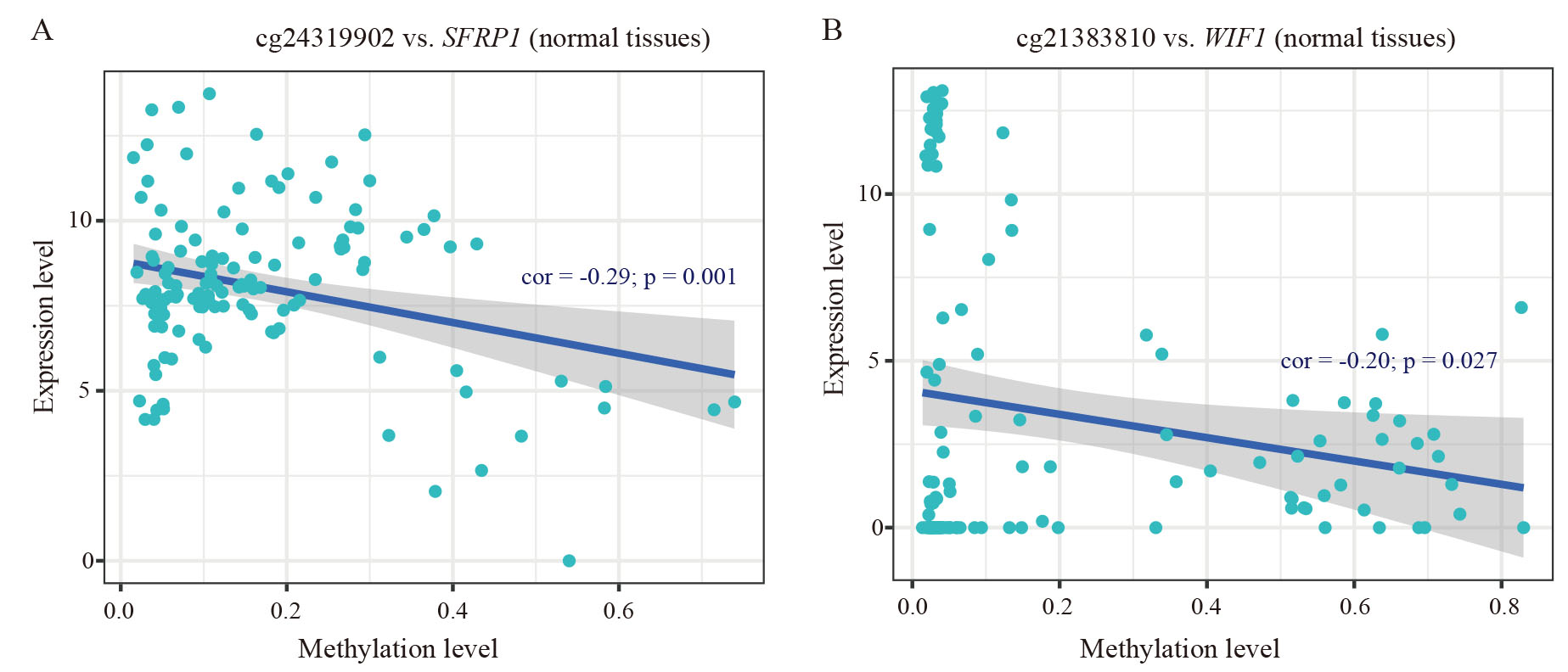


**Figure S6. Methylation-gene expression associations for the cg24319902-*SFRP1* and cg21383810-*WIF1* pairs in normal tissues.**

**Table S1.** Information of hypo-DMC activating genes that presenting a significant association between high expression and good survival probability in ER-positive breast cancer.

| **CpG** | **DMC (cluster 2: ER-positive vs. cluster 1: ER-positive)** | | **Methylation: Survival analysis** | | **Methylation - expression correlation analysis** | | **Gene name** | **Expression: survival analysis** | |
| --- | --- | --- | --- | --- | --- | --- | --- | --- | --- |
|  | **log2FC** | **p value** | **HR (95% CI)** | **p value** | **correlation** | **p value** |  | **HR (95% CI)** | **p value** |
| cg22839075 | -2.03059 | 1.67E-76 | 0.16 (0.048-0.55) | 0.00085 | -0.41315 | 5.97E-19 | SYN2 | 0.31 (0.14-0.7) | 0.003 |
| cg20888142 | -1.4723 | 2.85E-75 | 0.24 (0.087-0.64) | 0.002 | -0.48289 | 3.27E-26 | SYN2 | 0.31 (0.14-0.7) | 0.003 |
| cg23882862 | -1.91728 | 1.04E-57 | 0.32 (0.13-0.77) | 0.0071 | -0.29227 | 8.14E-10 | C11orf92 | 0.36 (0.15-0.89) | 0.022 |
| cg00995327 | -1.62037 | 3.96E-55 | 0.19 (0.063-0.57) | 0.00095 | -0.37784 | 7.18E-16 | CHST2 | 0.31 (0.13-0.76) | 0.0071 |
| cg02781618 | -1.06111 | 8.23E-51 | 0.25 (0.097-0.64) | 0.002 | -0.27756 | 5.89E-09 | BEND4 | 0.29 (0.12-0.68) | 0.0025 |
| cg02700894 | -1.06044 | 3.37E-47 | 0.15 (0.052-0.45) | 8.50E-05 | -0.41997 | 1.37E-19 | SYN2 | 0.31 (0.14-0.7) | 0.003 |
| cg09957386 | -1.18656 | 5.30E-47 | 0.27 (0.1-0.72) | 0.005 | -0.45608 | 3.20E-23 | AKR1B1 | 0.45 (0.2-1) | 0.047 |
| cg03217795 | -1.36143 | 5.72E-46 | 0.32 (0.14-0.74) | 0.0052 | -0.45633 | 3.01E-23 | PRKCB | 0.22 (0.098-0.51) | 0.00011 |
| cg22802813 | -1.0403 | 9.95E-45 | 0.33 (0.13-0.82) | 0.012 | -0.44866 | 1.94E-22 | USP44 | 0.43 (0.19-1) | 0.044 |
| cg24657817 | -1.44659 | 1.07E-43 | 0.27 (0.11-0.63) | 0.0013 | -0.26156 | 4.46E-08 | BEND4 | 0.29 (0.12-0.68) | 0.0025 |
| cg21079345 | -1.24339 | 4.36E-43 | 0.3 (0.12-0.75) | 0.0062 | -0.45743 | 2.29E-23 | AKR1B1 | 0.45 (0.2-1) | 0.047 |
| cg23011597 | -1.07198 | 5.37E-43 | 0.27 (0.11-0.64) | 0.0014 | -0.44229 | 8.78E-22 | KCNH8 | 0.46 (0.21-1) | 0.048 |
| cg10886442 | -1.43712 | 3.20E-42 | 0.13 (0.029-0.54) | 0.00098 | -0.37979 | 4.96E-16 | CHST2 | 0.31 (0.13-0.76) | 0.0071 |
| cg01398050 | -1.81524 | 3.52E-41 | 0.31 (0.13-0.76) | 0.0072 | -0.38419 | 2.13E-16 | KCNH8 | 0.46 (0.21-1) | 0.048 |
| cg09661010 | -1.04177 | 3.95E-41 | 0.35 (0.15-0.82) | 0.012 | -0.3497 | 1.14E-13 | SPOCK2 | 0.25 (0.098-0.64) | 0.0017 |
| cg23749856 | -1.0738 | 4.74E-41 | 0.35 (0.15-0.81) | 0.011 | -0.36275 | 1.16E-14 | CHST2 | 0.31 (0.13-0.76) | 0.0071 |
| cg19113375 | -1.30064 | 6.15E-40 | 0.37 (0.16-0.85) | 0.015 | -0.31944 | 1.55E-11 | NCRNA00092 | 0.25 (0.099-0.63) | 0.0014 |
| cg07399369 | -1.29337 | 9.44E-40 | 0.26 (0.098-0.71) | 0.0043 | -0.3901 | 6.73E-17 | CHST2 | 0.31 (0.13-0.76) | 0.0071 |
| cg14306956 | -1.62653 | 4.26E-39 | 0.39 (0.17-0.88) | 0.02 | -0.22882 | 1.87E-06 | PAX5 | 0.37 (0.16-0.83) | 0.012 |
| cg19046725 | -1.01699 | 4.30E-39 | 0.37 (0.16-0.86) | 0.017 | -0.35711 | 3.15E-14 | LEPREL1 | 0.42 (0.18-0.98) | 0.038 |
| cg02215070 | -1.30417 | 5.50E-39 | 0.28 (0.11-0.67) | 0.0024 | -0.42224 | 8.36E-20 | AKR1B1 | 0.45 (0.2-1) | 0.047 |
| cg14445814 | -1.272 | 1.15E-38 | 0.27 (0.11-0.69) | 0.0034 | -0.37865 | 6.16E-16 | DBC1 | 0.36 (0.15-0.85) | 0.015 |
| cg24668089 | -1.4322 | 1.64E-38 | 0.41 (0.17-0.99) | 0.041 | -0.38095 | 3.97E-16 | KCNH8 | 0.46 (0.21-1) | 0.048 |
| cg03308628 | -1.19757 | 1.66E-37 | 0.35 (0.15-0.82) | 0.012 | -0.33205 | 2.13E-12 | USP44 | 0.43 (0.19-1) | 0.044 |
| cg05347878 | -1.86935 | 3.32E-37 | 0.33 (0.14-0.81) | 0.011 | -0.2869 | 1.70E-09 | NCRNA00092 | 0.25 (0.099-0.63) | 0.0014 |
| cg18011364 | -1.56693 | 1.23E-36 | 0.31 (0.12-0.78) | 0.0089 | -0.31498 | 3.05E-11 | RELN | 0.37 (0.16-0.88) | 0.019 |
| cg12845520 | -1.57801 | 1.85E-36 | 0.46 (0.21-1) | 0.044 | -0.36741 | 4.98E-15 | C8orf47 | 0.42 (0.17-1) | 0.048 |
| cg13997864 | -1.23311 | 2.39E-36 | 0.31 (0.13-0.74) | 0.0051 | -0.55165 | 3.27E-35 | ITPRIPL1 | 0.38 (0.16-0.86) | 0.016 |
| cg21517947 | -1.66496 | 2.58E-36 | 0.18 (0.061-0.52) | 0.00035 | -0.38902 | 8.33E-17 | SFRP1 | 0.21 (0.08-0.57) | 0.00072 |
| cg08406370 | -1.46588 | 2.88E-36 | 0.42 (0.18-0.94) | 0.03 | -0.34959 | 1.16E-13 | PRKCB | 0.22 (0.098-0.51) | 0.00011 |
| cg10406295 | -1.1622 | 9.16E-36 | 0.1 (0.023-0.45) | 0.00022 | -0.41084 | 9.74E-19 | SFRP1 | 0.21 (0.08-0.57) | 0.00072 |
| cg18500192 | -1.44448 | 1.21E-35 | 0.38 (0.18-0.83) | 0.012 | -0.36874 | 3.91E-15 | KCNH8 | 0.46 (0.21-1) | 0.048 |
| cg17881007 | -1.21493 | 8.87E-35 | 0.45 (0.21-0.97) | 0.036 | -0.31855 | 1.77E-11 | WDR86 | 0.33 (0.12-0.91) | 0.024 |
| cg01108118 | -1.08432 | 4.10E-34 | 0.32 (0.14-0.76) | 0.0066 | -0.37753 | 7.61E-16 | IRF4 | 0.27 (0.11-0.65) | 0.0019 |
| cg08939095 | -1.65528 | 1.02E-33 | 0.26 (0.11-0.62) | 0.0011 | -0.27058 | 1.45E-08 | BNC1 | 0.35 (0.16-0.76) | 0.0052 |
| cg11542224 | -1.7416 | 1.45E-32 | 0.17 (0.057-0.48) | 0.00017 | -0.35176 | 7.99E-14 | TOX | 0.37 (0.16-0.87) | 0.018 |
| cg08774368 | -1.23664 | 1.55E-32 | 0.24 (0.097-0.61) | 0.001 | -0.40969 | 1.24E-18 | CHST2 | 0.31 (0.13-0.76) | 0.0071 |
| cg04092762 | -2.22459 | 2.81E-32 | 0.32 (0.13-0.76) | 0.0072 | -0.28665 | 1.76E-09 | LBH | 0.27 (0.11-0.68) | 0.0032 |
| cg19595234 | -1.64621 | 3.67E-32 | 0.16 (0.063-0.43) | 3.10E-05 | -0.23981 | 5.66E-07 | TOX2 | 0.37 (0.16-0.86) | 0.016 |
| cg17222500 | -1.21334 | 5.70E-32 | 0.15 (0.051-0.44) | 6.70E-05 | -0.38255 | 2.93E-16 | SLC6A15 | 0.18 (0.052-0.62) | 0.0025 |
| cg03108229 | -1.27568 | 1.59E-31 | 0.34 (0.14-0.81) | 0.01 | -0.2331 | 1.18E-06 | GLB1L3 | 0.32 (0.12-0.89) | 0.021 |
| cg24335138 | -1.6043 | 1.97E-31 | 0.35 (0.15-0.84) | 0.014 | -0.23035 | 1.59E-06 | ALX4 | 0.36 (0.15-0.85) | 0.015 |
| cg17816908 | -1.2053 | 2.93E-31 | 0.19 (0.064-0.55) | 6.00E-04 | -0.39308 | 3.73E-17 | SFRP1 | 0.21 (0.08-0.57) | 0.00072 |
| cg16439198 | -1.45873 | 3.03E-31 | 0.18 (0.07-0.46) | 7.10E-05 | -0.22216 | 3.76E-06 | CYP1B1 | 0.39 (0.15-0.99) | 0.04 |
| cg21383810 | -1.20767 | 5.59E-31 | 0.29 (0.11-0.8) | 0.011 | -0.29651 | 4.51E-10 | WIF1 | 0.25 (0.091-0.66) | 0.0027 |
| cg08858437 | -1.32061 | 6.89E-30 | 0.27 (0.098-0.72) | 0.0054 | -0.32594 | 5.63E-12 | CHST2 | 0.31 (0.13-0.76) | 0.0071 |
| cg24319902 | -1.0359 | 8.94E-30 | 0.27 (0.1-0.73) | 0.0055 | -0.44044 | 1.35E-21 | SFRP1 | 0.21 (0.08-0.57) | 0.00072 |
| cg19910780 | -1.83054 | 2.80E-29 | 0.23 (0.084-0.61) | 0.0013 | -0.27555 | 7.65E-09 | PRKCQ | 0.27 (0.12-0.64) | 0.0013 |
| cg19457477 | -1.1224 | 4.66E-29 | 0.41 (0.17-0.96) | 0.035 | -0.29357 | 6.80E-10 | MAP4K1 | 0.25 (0.097-0.64) | 0.0017 |
| cg02504416 | -1.3288 | 1.01E-28 | 0.35 (0.15-0.83) | 0.013 | -0.25379 | 1.14E-07 | ALX4 | 0.36 (0.15-0.85) | 0.015 |
| cg11667451 | -1.49995 | 1.49E-28 | 0.32 (0.14-0.75) | 0.0062 | -0.27154 | 1.28E-08 | TOX2 | 0.37 (0.16-0.86) | 0.016 |
| cg14557534 | -1.27286 | 1.86E-28 | 0.35 (0.15-0.86) | 0.017 | -0.20258 | 2.58E-05 | EOMES | 0.32 (0.13-0.76) | 0.0066 |
| cg26745222 | -1.27282 | 5.78E-28 | 0.32 (0.13-0.76) | 0.0067 | -0.2337 | 1.11E-06 | TOX2 | 0.37 (0.16-0.86) | 0.016 |
| cg16697214 | -1.34976 | 7.18E-28 | 0.36 (0.16-0.81) | 0.01 | -0.28788 | 1.49E-09 | IKZF1 | 0.33 (0.13-0.83) | 0.013 |
| cg11638200 | -1.43959 | 1.50E-27 | 0.35 (0.13-0.9) | 0.022 | -0.23115 | 1.46E-06 | PRKCQ | 0.27 (0.12-0.64) | 0.0013 |
| cg21692846 | -1.49451 | 6.06E-27 | 0.44 (0.21-0.9) | 0.021 | -0.22509 | 2.77E-06 | GLB1L3 | 0.32 (0.12-0.89) | 0.021 |
| cg24158594 | -1.33573 | 6.25E-27 | 0.33 (0.15-0.72) | 0.0035 | -0.2804 | 4.06E-09 | TOX | 0.37 (0.16-0.87) | 0.018 |
| cg22337605 | -1.70692 | 1.51E-26 | 0.31 (0.13-0.74) | 0.0051 | -0.29068 | 1.01E-09 | TNFRSF1B | 0.4 (0.18-0.9) | 0.022 |
| cg17228900 | -1.26932 | 1.86E-26 | 0.29 (0.12-0.69) | 0.003 | -0.32766 | 4.30E-12 | IRF4 | 0.27 (0.11-0.65) | 0.0019 |
| cg24337786 | -1.01865 | 1.95E-25 | 0.29 (0.12-0.73) | 0.0051 | -0.27561 | 7.58E-09 | MAP4K1 | 0.25 (0.097-0.64) | 0.0017 |
| cg18607529 | -1.75847 | 7.44E-25 | 0.4 (0.18-0.87) | 0.017 | -0.23144 | 1.42E-06 | IKZF1 | 0.33 (0.13-0.83) | 0.013 |
| cg09813525 | -1.51434 | 1.52E-24 | 0.37 (0.16-0.86) | 0.016 | -0.23704 | 7.70E-07 | PCDH8 | 0.4 (0.16-0.99) | 0.041 |
| cg26734696 | -1.60085 | 1.53E-24 | 0.36 (0.15-0.86) | 0.016 | -0.27485 | 8.38E-09 | HS3ST4 | 0.31 (0.12-0.8) | 0.011 |
| cg08862890 | -1.00428 | 1.99E-24 | 0.38 (0.18-0.82) | 0.01 | -0.20619 | 1.83E-05 | DOCK2 | 0.35 (0.16-0.79) | 0.0085 |
| cg13467254 | -1.35552 | 2.87E-24 | 0.23 (0.086-0.63) | 0.0017 | -0.30578 | 1.19E-10 | TOX | 0.37 (0.16-0.87) | 0.018 |
| cg16132520 | -1.0522 | 4.49E-24 | 0.23 (0.091-0.59) | 0.00089 | -0.28187 | 3.34E-09 | AKR1B1 | 0.45 (0.2-1) | 0.047 |
| cg18944047 | -1.18204 | 9.65E-24 | 0.13 (0.045-0.38) | 9.60E-06 | -0.27863 | 5.12E-09 | KIF19 | 0.29 (0.11-0.73) | 0.0049 |
| cg00862116 | -1.27229 | 1.00E-22 | 0.35 (0.14-0.86) | 0.018 | -0.23819 | 6.78E-07 | ABCB1 | 0.43 (0.21-0.9) | 0.021 |
| cg11018723 | -1.2884 | 1.09E-22 | 0.42 (0.18-0.96) | 0.034 | -0.32905 | 3.45E-12 | RUNX3 | 0.36 (0.15-0.83) | 0.013 |
| cg08318212 | -1.08981 | 1.15E-22 | 0.47 (0.22-1) | 0.045 | -0.27103 | 1.37E-08 | EDN3 | 0.44 (0.19-0.99) | 0.041 |
| cg09507526 | -1.15158 | 1.85E-22 | 0.23 (0.092-0.58) | 0.00077 | -0.28055 | 3.97E-09 | PRKCB | 0.22 (0.098-0.51) | 0.00011 |
| cg09053081 | -1.07427 | 1.88E-22 | 0.45 (0.21-0.99) | 0.042 | -0.40992 | 1.18E-18 | CD40 | 0.37 (0.16-0.84) | 0.013 |
| cg19954537 | -1.24894 | 4.53E-22 | 0.39 (0.16-0.95) | 0.032 | -0.52372 | 2.62E-31 | ARID5A | 0.3 (0.12-0.75) | 0.0063 |
| cg06864853 | -1.25646 | 6.28E-22 | 0.41 (0.18-0.89) | 0.021 | -0.24969 | 1.84E-07 | AKR1B1 | 0.45 (0.2-1) | 0.047 |
| cg25537217 | -1.15608 | 8.60E-22 | 0.43 (0.19-0.97) | 0.037 | -0.21207 | 1.04E-05 | WIPF1 | 0.36 (0.15-0.87) | 0.018 |
| cg06164660 | -1.0225 | 2.80E-21 | 0.41 (0.17-0.96) | 0.033 | -0.28939 | 1.21E-09 | TMEM132C | 0.27 (0.11-0.69) | 0.0032 |
| cg27496650 | -1.40674 | 9.70E-20 | 0.29 (0.12-0.73) | 0.0048 | -0.32166 | 1.10E-11 | TOX | 0.37 (0.16-0.87) | 0.018 |
| cg22500428 | -1.72974 | 3.33E-19 | 0.43 (0.19-0.95) | 0.032 | -0.36917 | 3.61E-15 | CCND2 | 0.38 (0.17-0.85) | 0.015 |
| cg02797548 | -1.50448 | 3.81E-18 | 0.4 (0.16-0.99) | 0.04 | -0.20809 | 1.53E-05 | EOMES | 0.32 (0.13-0.76) | 0.0066 |
| cg01367393 | -1.58914 | 4.67E-17 | 0.14 (0.04-0.46) | 0.00019 | -0.26387 | 3.35E-08 | HAPLN3 | 0.26 (0.096-0.73) | 0.0057 |
| cg12505146 | -1.61201 | 5.44E-17 | 0.44 (0.2-0.95) | 0.033 | -0.25097 | 1.58E-07 | KIF19 | 0.29 (0.11-0.73) | 0.0049 |
| cg24966702 | -1.08041 | 5.32E-10 | 0.39 (0.17-0.91) | 0.023 | -0.26063 | 4.99E-08 | DACT2 | 0.27 (0.11-0.65) | 0.0017 |
